# Prevalence of self-medication during COVID-19 pandemic: A systematic review and meta-analysis

**DOI:** 10.3389/fpubh.2022.1041695

**Published:** 2022-11-03

**Authors:** Golnesa Kazemioula, Shayan Golestani, Seyed Mohammad Amin Alavi, Forough Taheri, Reza Ghanei Gheshlagh, Mohammad Hassan Lotfalizadeh

**Affiliations:** ^1^Department of Medical Genetics, School of Medicine, Tehran University of Medical Sciences, Tehran, Iran; ^2^Department of Oral and Maxillofacial Surgery, Dental School, Islamic Azad University, Isfahan, Iran; ^3^Faculty of Medicine, Ahvaz Jundishapur University of Medical Sciences, Ahvaz, Iran; ^4^Department of Medical Genetics, Faculty of Medicine, Isfahan University of Medical Sciences, Isfahan, Iran; ^5^Social Determinants of Health Research Center, Research Institute for Health Development, Kurdistan University of Medical Sciences, Sanandaj, Iran; ^6^Board Certificate Oral and Maxillofacial Radiologist, North Khorasan University of Medical Sciences (NKUMS), Bojnurd, Iran

**Keywords:** self-medication, prevalence, systematic review, COVID-19, meta-analysis

## Abstract

**Background:**

The COVID-19 pandemic and restrictions on travel and quarantine measures made people turn to self-medication (SM) to control the symptoms of their diseases. Different studies were conducted worldwide on different populations, and their results were different. Therefore, this global systematic review and meta-analysis was conducted to estimate the pooled prevalence of self-medication.

**Methods:**

In this systematic review and meta-analysis, databases of Scopus, PubMed, Embase, and Web of Science were searched without a time limit. All eligible observational articles that reported self-medication during the COVID-19 pandemic were analyzed. Heterogeneity among the studies was assessed using Cochran's Q test and I^2^ statistics. A random-effects model was used to estimate the pooled prevalence of self-medication. The methodological quality of the articles was evaluated with the Newcastle-Ottawa Scale.

**Results:**

Fifty-six eligible studies were reviewed. The pooled prevalence of self-medication was 48.6% (95% CI: 42.8–54.3). The highest and lowest prevalence of self-medication was in Asia (53%; 95% CI: 45–61) and Europe (40.8%; 95% CI: 35–46.8). Also, the highest and lowest prevalence of self-medication was related to students (54.5; 95% CI: 40.8–68.3) and healthcare workers (32.5%; 16–49). The prevalence of self-medication in the general population (48.8%; 40.6–57) and in patients with COVID-19 (41.7%; 25.5–58). The prevalence of self-medication was higher in studies that collected data in 2021 than in 2020 (51.2 vs. 48%). Publication bias was not significant (*p* = 0.320).

**Conclusion:**

During the COVID-19 pandemic, self-medication was highly prevalent, so nearly half of the people self-medicated. Therefore, it seems necessary to provide public education to control the consequences of self-medication.

## Introduction

On January 30, 2020, the World Health Organization (WHO) declared a state of public health emergency due to the emergence of COVID-19 disease. Six months later, ~20 million cases and 700,000 deaths were reported worldwide (1). People resorted to self-medication (SM) due to the fear of contracting COVID-19, misinformation, and low access to health services. With people confined to their homes, the Internet was the only source of information they had access to. Also, when the hospitals were filled with patients, people were afraid to go to the hospitals and started self-medication (2).

Self-medication refers to choosing and using drugs to treat self-diagnosed symptoms and diseases without consulting a doctor (3). Self-medication includes the purchase and use of over-the-counter (OTC) medications, prescription-only medications (POMs), and leftover medication out of recommended (4). Self-medication leads to resource waste, increased pathogens resistance, and antibiotic resistance (3, 5). Also, self-medication is associated with incorrect dosage, incorrect route of administration, long-term use, improper storage, drug interactions, polypharmacy, and the risk of dependence and abuse, so it has become a serious public health problem worldwide (6, 7). In most cases, the feeling of the mildness of the disease and no need to consult a doctor, previous pleasant experiences with self-treatment, the feeling of being able to self-care, and the lack of availability of a doctor increase self-treatment (4). Furthermore, socio-economic factors, lifestyle, sources of medical information, access to medicines, and the potential of managing some diseases through self-care are related to the continuous increase of self-treatment worldwide (7, 8). On the other hand, self-treatment reduces the economic burden on patients, the high cost of hospital treatments, and the pressure on health care systems, limiting the number of hospital visits (6, 9). Because self-treatment starts with self-diagnosis, the probability of its being incorrect is high, and even a correct diagnosis can be associated with the wrong treatment choice. Also, the average consumers do not know if they are in a particular group with significant side effects of medicine or not; they are unaware of drug contraindications. Sometimes, the patients take the same active ingredient with a different name, and there is a risk of double medication or harmful interactions. Sometimes there is a risk of the wrong prescription (e.g., intravenous instead of intramuscular) (10).

Different studies that have investigated the prevalence of self-medication in the COVID-19 pandemic around the world have reported different results. Knowing the prevalence of self-medication in this pandemic can provide health policymakers and researchers with helpful information. Therefore, the present study was conducted to estimate the overall prevalence of self-medication in the COVID-19 pandemic.

## Methods

This systematic review and meta-analysis, which sought to estimate the pooled prevalence of self-medication in endemic COVID-19 globally, was conducted according to the Preferred Reporting Items for Systematic Reviews and Meta-Analyses (PRISMA) guidelines (11). The PRISMA checklist is attached as a Supplementary File 1. The protocol of this systematic review was not registered in Cochrane and PROSPERO.

### Search strategy

We searched PubMed, Scopus, Web of Science, and Embase databases from January 2020 to May 2022 using the following terms: (“Self Medication” OR “Self Medicat^*^” OR “SelfMedicat^*^”) AND (“COVID-19” OR “SARS-CoV-2” OR “COVID 19” OR “2019-nCoV” OR “Coronavirus Disease-19” OR “SARS CoV 2” OR “2019 Novel Coronavirus” OR “Wuhan Coronavirus” OR “SARS Coronavirus 2” OR “Wuhan Seafood Market Pneumonia Virus”). Databases were searched in June 2022. To access more articles, the reference list of selected articles was reviewed. Further details are provided in Table 1.

**Table 1:** The search strategy.

PubMed	(“Self Medication”[Mesh] OR “Self Medicat*”[tiab] OR “Self-Medicat*”[tiab] OR “SelfMedicat*”[tiab]) AND (“COVID-19”[Mesh] OR “SARS-CoV-2”[Mesh] OR “COVID 19”[tiab] OR “2019-nCoV”[tiab] OR “Coronavirus Disease-19”[tiab] OR “SARS CoV 2”[tiab] OR “2019 Novel Coronavirus”[tiab] OR “Wuhan Coronavirus”[tiab] OR “SARS Coronavirus 2”[tiab] OR “Wuhan Seafood Market Pneumonia Virus”[tiab] )
Scopus	TITLE-ABS-KEY(“Self Medicat*” OR “Self-Medicat*” OR “SelfMedicat*”) AND TITLE-ABS-KEY (“COVID-19” OR “SARS-CoV-2” OR “COVID 19” OR “2019-nCoV” OR “Coronavirus Disease-19” OR “SARS CoV 2” OR “2019 Novel Coronavirus” OR “Wuhan Coronavirus” OR “SARS Coronavirus 2” OR “Wuhan Seafood Market Pneumonia Virus”)
Web of science	TS=(“Self Medicat*” OR “Self-Medicat*” OR “SelfMedicat*”) AND TS=(“COVID-19” OR “SARS-CoV-2” OR “COVID 19” OR “2019-nCoV” OR “Coronavirus Disease-19” OR “SARS CoV 2” OR “2019 Novel Coronavirus” OR “Wuhan Coronavirus” OR “SARS Coronavirus 2” OR “Wuhan Seafood Market Pneumonia Virus”) Timespan: All years. Indexes: SCI-EXPANDED, SSCI, A&HCI, ESCI.
Embase	('Self Medicat*':ta,ab,kw OR 'Self-Medicat*':ta,ab,kw OR 'SelfMedicat*':ta,ab,kw) AND ('COVID-19':ta,ab,kw OR 'SARS-CoV-2':ta,ab,kw OR 'COVID 19':ta,ab,kw OR '2019-nCoV':ta,ab,kw OR 'Coronavirus Disease-19':ta,ab,kw OR 'SARS CoV 2':ta,ab,kw OR '2019 Novel Coronavirus':ta,ab,kw OR 'Wuhan Coronavirus':ta,ab,kw OR 'SARS Coronavirus 2':ta,ab,kw OR 'Wuhan Seafood Market Pneumonia Virus':ta,ab,kw)

### Selection of studies and data extraction

The inclusion criteria were: observational studies reporting data on the prevalence or frequency of self-medication during the COVID-19 pandemic and published in English. Exclusion criteria included unrelated studies, interventional studies, review articles, theses, case reports, and repeated articles. According to the inclusion and exclusion criteria, the authors independently read the title and abstract of all articles and separated the relevant ones. In the next step, the authors reviewed the full text of these articles, and essential information such as the first author's name, year of publication, sample size, type of study, country, target group, mean age, and time of data collection were recorded in a pre-prepared form. Any disagreement was resolved by consultation and discussion.

### Quality assessment

We used the Newcastle-Ottawa scale to check the methodological quality and assess the bias of the articles, which included seven items and three dimensions of selection, comparison, and result. These dimensions were assigned 5, 2, and 3 stars, respectively. The selection dimension with four items evaluates the target population, sample size estimation, non-response rate, and measurement tool. The first three items can be assigned up to one star and the fourth item up to two stars. The comparable dimension evaluates the use of the control group and can get up to two stars. The result dimension has two items evaluating the result (two stars) and statistical tests (one star). High-quality articles were defined as ≥4 stars (12). The bias assessment was checked by two authors independently, and any disagreements were resolved through consultation.

### Meta-analysis

STATA version 16 software was used for data analysis. We used the binomial distribution to combine the selected studies. To show the general prevalence of self-medication from the Forest plot and to examine the heterogeneity among the selected studies, we used the I^2^ index and Cochran's Q test. We considered the I^2^ level >75% as high heterogeneity. Because the I^2^ index in this study was more than 75% and Cochran's Q test was significant, the random effects model was used to combine the selected studies. Subgroup analysis was performed based on the continent, target population, and year of data collection. Also, a meta-regression test was used to investigate the relationship between the prevalence of self-medication and the mean age of the participants. The publication bias of the studies was evaluated using a funnel plot based on Egger's regression.

## Results

**Figure 1:**
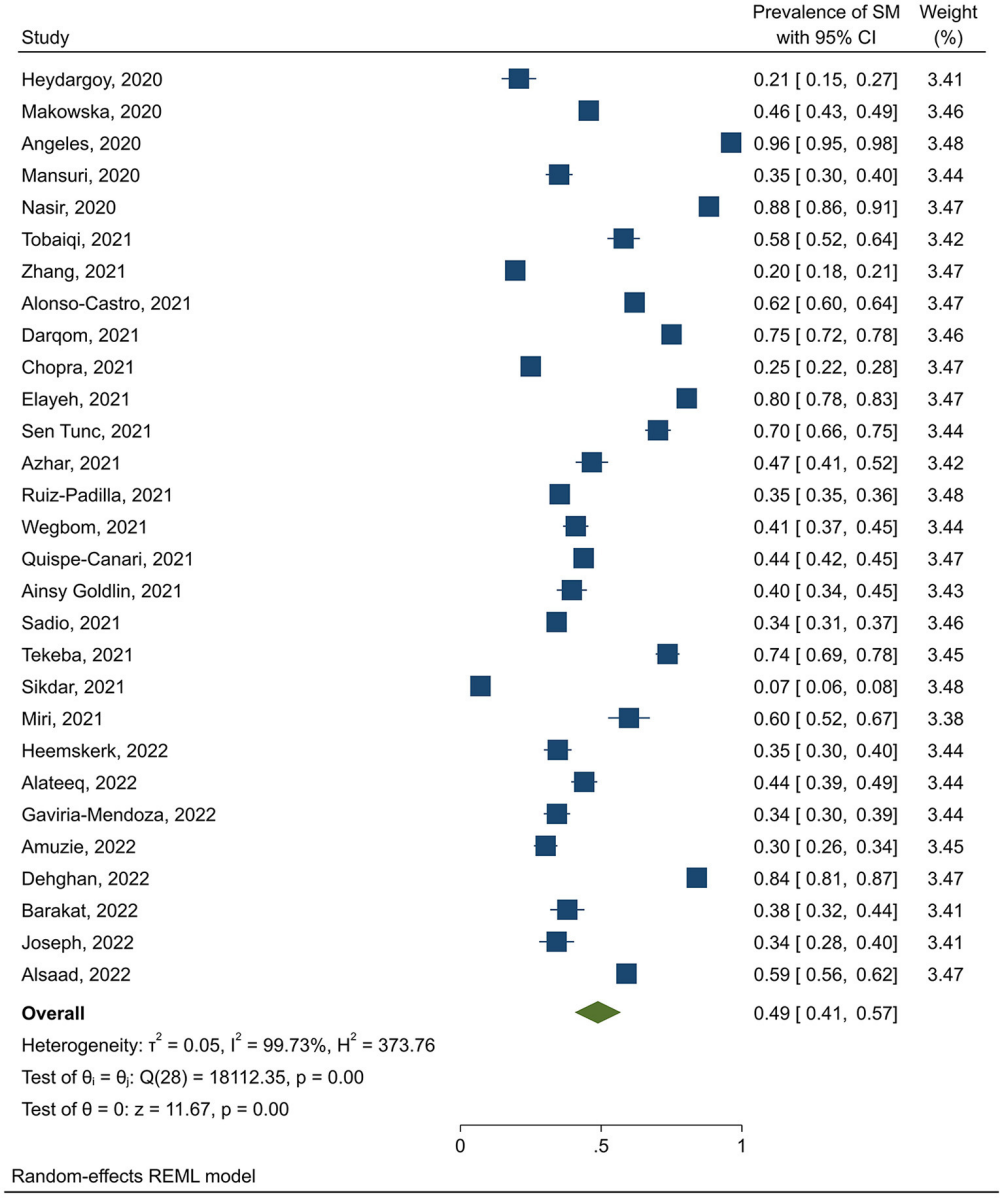
The process of screening and selecting the articles based on the PRISMA guidelines.

In the initial search, 674 articles were retrieved, of which 359 were removed due to duplication. Then, the authors independently reviewed the remaining articles' titles and abstracts. After screening the articles based on the inclusion and exclusion criteria, 186 articles were excluded. The full text of six articles could not be retrieved. The full text of the remaining 123 articles was reviewed. Sixty-seven articles were excluded due to not reporting the prevalence of self-medication or incomplete reports. Finally, 56 articles with a sample size of 56,063 subjects were included in the analysis (Figure 1).

In terms of quality, 32 articles had moderate quality, and 24 studies had excellent quality (Supplementary File 2). Twenty-six studies were published in 2022, 24 in 2021, and 6 in 2020. Twelve articles published in 2022, 14 published in 2021, and two published in 2020 were high quality. Most of the studies were conducted in the Asian continent (*n* = 31) and on the general population (*n* = 29). Also, the data of 31 studies were collected in 2020. Most studies were conducted in three countries: India (*n* = 11), Saudi Arabia (6 studies), and Nigeria (6 studies) (Table 2). The pooled prevalence of self-medication in these three countries was 45% (95% CI: 35–55), 57% (95% CI: 43–71), and 43.5% (95% CI: 26.5–60.5), respectively.

**Table 2:** The characteristics of included studies.

**First author**	**Year**	**Sample size**	**Country**	**Mean age**	**Target group**	**Prevalence (%)**	**Date of data collection**	**Self-medication agent**	**Findings**	**Quality**
Alsaad (7)	2022	1,226	Saudi Arabia	–	General population	59	January to February 2021	–	The highest prevalence of SM was in women, workers in health departments—excluding physicians and pharmacists- and people with chronic diseases.	High
Kashyap (13)	2022	326	India	–	Medical students	29.8	–	Supplementary medicines	About one-third of the participants stated they self-medicated to prevent or treat COVID-19. Also, the most common reason for SM was easy to access.	High
Malik (14)	2022	451	Pakistan	–	Dental patients	86.2	September to December 2020	Pain relievers and Antibiotics	The most common causes of SM were toothache (56.8%), and tooth sensitivity (37.5%).	Moderate
Likhar (15)	2022	394	India	–	Medical students	73.8	–	–	The main reason for SM was convenience (23.09%) and the intention of getting quick relief (21.06%).	Moderate
Aitafo (6)	2022	220	Nigeria	–	HCWs	15.9	January to March 2022	Vitamin C, Zinc, Azithromycin, and anti-malarial	The main reason for SM was the fear of getting infected following contact with suspected or confirmed cases of COVID-19	High
Alavi Namvar (16)	2022	306	Iran	34.9	Dental patients	53.9	October 2020 and April 2021	Ibuprofen; Acetaminophen; Novafen; Mefenamic acid; Amoxicillin; Metronidazole; Penicillin; and Salt and water mouthwash	Low education level was associated with SM. The most common problem for SM was toothache.	Moderate
Barakat (17)	2022	245	Egypt	–	General population	38	–	–	The most predictors of SM were internet use for getting medical information (OR = 2.1, *p* = 0.02), lack of health education about COVID-19 (OR = 2.1, *p* = 0.03), and younger age (OR = 0.9, *p* = 0.03).	Moderate
Yasmin (18)	2022	489	Pakistan	–	Medical students	83	January to February 2021	Paracetamol and multivitamins	Most SM was reported in women, third-year medical students and people in good self-reported health.	High
Acharya (3)	2022	383	USA	–	Medical students and staff	50.4	November 2021	Paracetamol, Vitamin C; Zinc; Multivitamins; Vitamin D; Azithromycin; Cough syrup; and Ibuprofen	More than half of the participants purchased the medicines directly from the pharmacy.	Moderate
Alateeq (19)	2022	443	Saudi Arabia	37.56	General population	44	July to August 2021	Dietary Supplements	The predictors of dietary supplement use were insomnia and a history of mental health disorder diagnosis.	High
Amuzie (20)	2022	469	Nigeria	39.9 ± 13.5	General population	30.3	October to November 2021	Herbal products; Antimalarial; Vitamin Supplements; Azithromycin; Ivermectin; Analgesics; Calcium Supplements; Hydroxychloroquine; Ciprofloxacin	Older age (AOR = 1.87), primary education (AOR = 2.15) and perception of cost (AOR = 2.29) were predictors of self-medication.	High
Bello (21)	2022	356	Nigeria	20.34	Students	65.4	May to August 2020	Paracetamol; Tramadol; Cold syrup; Vitamin C; Herbs; Anti-Malaria; Anti-Diarrhea; Piriton; Slimming Pills and Teas; Food Supplements; and Hydroxychloroquin	Experience of COVID-19 symptoms significantly predicted SM. The prevalence of SM was not significantly different between male and female undergraduate students.	High
Gaviria-Mendoza (22)	2022	397	Columbia	31	General population	34.3	June to September 2020	Acetaminophen; Antihistamines; Vitamins; and Antibiotics	The reasons for SM to prevent COVID-19 were: distrust of personnel and health centers (OR = 10.4; *p* = 0.013) and fear of being fined for leaving home (OR = 7.29; *p* = 0.026).	Moderate
Dehghan (23)	2022	782	Iran	–	General population	84	April to August 2021	Nutritional supplements	SM was associated with sex, having children, place of residence, and COVID-19 status.	High
Gerbier (24)	2022	5,210	European countries	–	Pregnant and Postpartum Women	41.4	June to August 2021	Paracetamol; Cetirizine; Omeprazole; Acetylsalicylic acid; Lactulose; Metoclopramide; Salbutamol; Levothyroxine sodium; and Antibiotics	Analgesics were the most commonly used drugs. Antihistamines and drugs for gastric-related disorders were the most commonly used drugs. Also, NSAIDs and antihistamines were the most commonly used drugs in women.	High
González-González (25)	2022	284	Mexico	–	University Students	26	2020	Herbal; Medicinal Drugs; Vitamins; and Chlorine dioxide	About 20% of the participants had self-medicated to prevent COVID-19. The prevalence of SM did not differ between men and women.	Moderate
Gupta (26)	2022	170	India	38.6 ± 10.7	Students	57.7	August 2020	pain suppression; Antibiotics and anti-acid medications	Students with less educated tend to SM more than those with higher education (OR = 2.200, 95% CI = 1.116–4.336).	Moderate
Heemskerk (27)	2022	361	France	–	General population	34.6	November 2020 and January 2021	Vitamins; herbal or home remedies	A third of the participants had used SM to protect themselves against COVID-19 and boost their immune systems. Women were more likely than men to use over-the-counter medications, vitamins, and herbal/home remedies to protect themselves from COVID-19.	Moderate
Joseph (28)	2022	225	India	34.5 ± 15.2	General population	34.2	August 2021	Paracetamol	The most common symptom for which SM was performed was a cold, and the most common drug used was paracetamol. Participants with a history of self-medication among family members, relatives, or friends were more likely to self-medicate. The idea that SM is harmful was more in women than in men.	High
Kumari (29)	2022	57	India	46.3± 8.8	Faculty employed	50.8	–	Analgesics; Vitamins; Antibiotics; and Anti-allergic drugs	The most common reason for SM was that it was less costly. Doctors at work were the most common source of information. The most common symptom that caused SM was pain. The most common drug used was analgesics.	Moderate
Mahmoudi (30)	2022	450	Iran	–	COVID-19 patients	56.1	March to October 2020	Not reported	Not reported	Moderate
Odis (31)	2022	402	Nigeria	–	Outpatients	71.9	February 2021	Acetaminophen; Ibuprofen; Azithromycin; Penicillin; Antiretrovirals; and hydroxychloroquine	Older people used antiretroviral drugs more than other age groups.	Moderate
Okoye (32)	2022	669	Nigeria	35.6 ± 8.7	HCWs	36.3	March and April 2021	Ivermectin; Azithromycin; Vitamin C; Chloroquine; and zinc	Factors associated with self-medication were: older age, being a pharmacist, higher income, and previous COVID-19 test. Age > 44 years (AOR = 2.77) and previous COVID-19 test (AOR = 2.68) were predictors of SM.	High
Rojas-Miliano (33)	2022	166	Peru	–	Students	14.5	October to November 2020	Paracetamol; Aspirin; and Influenza drugs	SM was associated with the perception that SM is harmful to health (PR = 0.41; 95% CI = 0.20–0.84) and having a doctor as a source of medication information (PR = 0.46; CI = 0.21–0.99).	High
Toure (34)	2022	975	Guinea	–	Health centers staff	14.1	June 2021	Not reported	Fatigue (OR = 2.11), sore throat (OR = 1.89), loss of smell (OR = 4.64), and sore throat of a close person (OR = 2.32) were independently associated with SM.	Moderate
Vasquez-Elera (35)	2022	301	Peru	58.6 ± 16.4	COVID-19 patients	54.8	May to June 2020	Vermectin; Azithromycin; Corticosteroids; and NSAIDs	The frequency of SM in people between 30 and 59 years old was 2.53 times higher than in people between 18 and 29 years old. Also, male gender, dyslipidemia, smoking, and hepatic steatosis were related to SM.	Moderate
Zeng (36)	2021	70	China	–	COVID-19 patients	31.4	January to February 2020	Traditional Chinese medicine; Acetaminophen Levofloxacin; Antivirals; and Antibiotics	Not reported	Moderate
Saleem (37)	2021	520	Saudi Arabia	21.8 ± 1.9	Students	58	March to June 2020	Analgesics; Antibiotics; Antipyretics; Antihistamines; Antidiarrheal, Antiemetic; Antacid; Laxatives; Food supplements; and Vitamins	The most reasons for SM were: symptoms are minor, experience, saving time, pharmacist advice, and saving money.	High
Sadio (1)	2021	955	Togo	–	General population	34.2	April to May 2020	Vitamin C and traditional medicine	Female sex (AOR = 1.90), work in the health sector (AOR = 1.89), secondary education level (AOR = 2.28), and university education level (AOR = 5.11) were associated with SM.	High
Ruiz-Padilla (38)	2021	16,724	Mexico	–	General population	35.3	March to November 2020	Aspirin; Ibuprofen; Dexamethasone; Azithromycin, Ivermectin; Hydroxychloroquine; and Chloroquine	The factors associated with SM were age (18–25 years), female gender, low education level, low socioeconomic status, being married, unemployment, and the presence of comorbidity.	High
Mota (39)	2021	710	Brazil	–	HCWs	60.3	May to July 2020	Insomnia medication	About two-thirds of the total sample had some sleep-related complaints, 25.8% due to difficulty initiating sleep, 29.6% due to difficulty staying asleep, and 32.5% due to early morning waking.	Moderate
Zhang (40)	2021	2,217	Australia	–	General population	19.5	March 2020	Antibiotics	Age (OR = 0.89), gender (OR = 1.29), and education (OR = 1.13) were associated with antibiotic use for protection against COVID-19.	Moderate
Heshmatifar (41)	2021	342	Iran	66.2 ± 5.67	Elderly	55.5	–	Analgesics; vitamins; Anti-cold; and Antibiotics	The main factors related to SM were disease prevention, home quarantine, financial problems, previous experience of SM, and others' advice.	Moderate
Ainsy Goldlin (42)	2021	323	India	–	General population	39.6	June and July 2020	Hydroxychloroquine; Azithromycin; Ivermectin; Herbal preparations, Vitamins; and Minerals preparations	The main factors related to SM were COVID-19 spread and mortality, fear of visiting hospitals, time saving, lack of easy access to hospitals, easy availability of drugs in local pharmacies, trust in online information and being familiar with the drugs	Moderate
Alonso-Castro (43)	2021	2,100	Mexico	32.1± 13.6	General population	61.9	March and June 2020	Herbs	Female gender, age < 40 years, low education level, being single, unemployment, presence of mental illness, use of psychiatric drugs, and drug use were among the factors related to self-medication.	High
Azhar (44)	2021	290	Pakistan	–	General population	46.7	–	Analgesics; Hydroxychloroquinone; Azithromycin; and Ivermectin	The most frequent reason for SM was the unavailability of doctors.	Moderate
Chopra (45)	2021	1,100	India	–	General population	25	May 2020	Hydroxychloroquine; Herbal drugs; Vitamins; Antimicrobials; Antihistamines	SM was more common in women than men and in married people than single people.	Moderate
D'arqom (46)	2021	610	Indonesia	–	Women	75	July to December 2020	Anti-COVID medications; Vitamins; and Herbal Supplements	SM in housewives was related to education, and SM in working women was related to age and family income.	High
Faqihi (47)	2021	177	Saudi Arabia	20 ± 3	Nursing students	87	December 2019 to February 2020	Acetaminophen; Ibuprofen; Diclofenac Meloxicam; Aspirin; Azithromycin; Amoxicillin; Doxycycline; Metronidazole; and Ampicillin	The most common self-medication causes were headaches, menstrual pain, and fever. The main reason for self-medication was a lack of time to consult a doctor.	Moderate
Mir (48)	2021	168	India	–	General population	59.9	May 2021	Paracetamol; Azithromycin; Expectorants; Ivermectin; Doxycycline; Corticosteroids; Hydroxychloroquine	Not reported	Moderate
Islam (49)	2021	1,002	Bangladesh	34.7 ± 13.9	COVID-19 patients	24	September to October 2020	Not reported	Lower socioeconomic status and persistent symptoms of COVID-19 were associated with SM. The main reasons for SM were: insufficient local medical services, dissatisfaction with local health care services, cost of a consultation with doctors, and lack of time.	Moderate
Elayeh (50)	2021	1,179	Jordan	32.0 ± 12.5	General population	80.4	March to April 2021	Antihistamine; Cold preparations; Immune boosters; Omega-3; Propolis; Vitamins; Iron; Zink; Ibuprofen; Paracetamol; Magnesium	Female gender (OR = 1.603), working in the medical field (OR = 1.697), and history of COVID- 19 infection (OR = 2.026) were associated with SM.	High
Choudhary (51)	2021	100	India	36.94 ± 11.83	Dermatology patients	48	June 2020	Steroids; Antibiotics; Antifungals; Immunomodulators; Antihistamines; and Dithranol	Fear of infection from healthcare facilities and reduced access to healthcare facilities due to lockdown because of COVID-19 were associated with SM.	Moderate
Sen Tunc (52)	2021	389	Turkey	–	Parents	70.2	July to October 2020	Analgesics; antibiotics; mouthwashes; and herbal medicines	The main reason for SM was difficulty obtaining a dental consultation.	High
Sikdar (53)	2021	2,941	Bangladesh	–	General population	7.14	November to December 2020	Sedatives; Anxiolytics; and Anti-depressant drugs	SM was more in people over 35 years old and men than in other groups.	Moderate
Quispe-Cañari (54)	2021	3,792	Peru	–	General population	43.8	May to June 2020	Vitamin C; Traditional medicines; Chloroquine/Hydroxychloroquine	There was a relationship between age, the region where one lived, and job status with SM.	High
Tekeba (55)	2021	416	Ethiopia	24.3 ± 5.1	Pharmacy clients	73.6	June 2020	Painkillers; Antibiotics; Cough syrup; Antacid; and Oral contraceptive	SM was associated with age 18–24 years (AOR = 9.28), and 25–34 years (AOR = 3.54), current single status (AOR = 0.28), government employment (AOR = 0.31), and limited knowledge (AOR = 2.31).	High
Tobaiqi (56)	2021	281	Saudi Arabia	–	General population	58	July to September 2020	Laxatives; Antacid; Eye drops; Vitamins; Herbs; Antibiotics; Analgesics	The most common symptom that caused the use of SM was a headache. Also, the most used drug in SM was painkillers.	Moderate
Vinay (57)	2021	39	India	39.6 ± 14.1	Inflammatory Bowel Disease Patients	17.9	March to June 2021	Steroids	Not reported.	Moderate
Wegbom (58)	2021	461	Nigeria	42.2 ± 10.7	General population	41	June to July 2020	Hydroxychloroquine; Chloroquine; Erythromycin; Metronidazole; Herbal products; Ciprofloxacin; Vitamins	Male gender (OR = 0.79) and having sufficient knowledge about SM (OR = 0.64) were associated with SM.	High
Onchonga (59)	2020	379	Kenya	–	HCWs	36.2	–	Not reported	Physically active participants who worked during the day and were healthy were less likely to self-medicate.	High
Heydargoy (60)	2020	168	Iran	–	General population	20.8	–	Antibiotics	Not reported	Moderate
Makowska (61)	2020	1013	Poland	–	General population	45.6	June 2020	Not reported	Not reported	High
Mansuri (62)	2020	385	Saudi Arabia	–	General population	35.1	March to April 2020	Not reported	Not reported	Moderate
Nasir (63)	2020	626	Bangladesh	–	General population	88.3	April to May 2020	Ivermectin; Azithromycin; Montelukast; Calcium supplements; Doxycycline; Hydroxychloroquine	The most important symptoms that caused SM were fever, throat pain, and dry cough.	Moderate
de los Angeles (64)	2020	829	Ecuador	–	General population	96.2	–	Eucalyptus; Ginger	Not reported	Moderate

NSAIDs, non-steroidal anti-inflammatory drugs; OR, odds ratio; AOR, adjusted odds ratio; PR, prevalence ratio; CI, confidence interval.

**Figure 2:**
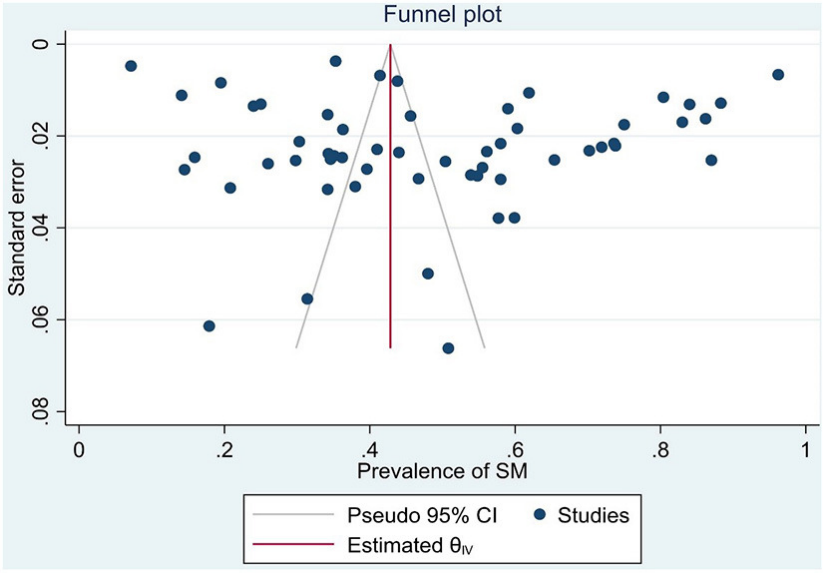
Prevalence of self-medication in the general population during the COVID-19 pandemic.

The pooled prevalence of self-medication was 49% (95% CI: 43–54). Also, the findings by continent showed that the highest and lowest prevalence of self-medication was in Asia (53%, 95% CI: 45–61) and Europe (40.8%, 95% CI: 35–46.8), respectively. The prevalence of self-medication in America (47.8%, 95% CI: 33.6–62) was higher than in Africa (41.5%, 95% CI: 29.5–53.5). By dividing the target population, the findings showed that the highest and lowest prevalence of self-treatment was related to students (54.5%, 95% CI: 40.8–68.3) and healthcare workers (35.5%, 95% CI: 16–49). The prevalence of self-medication in the general population was 49% (95% CI: 41–57) (Figure 2).

**Figure 3:**
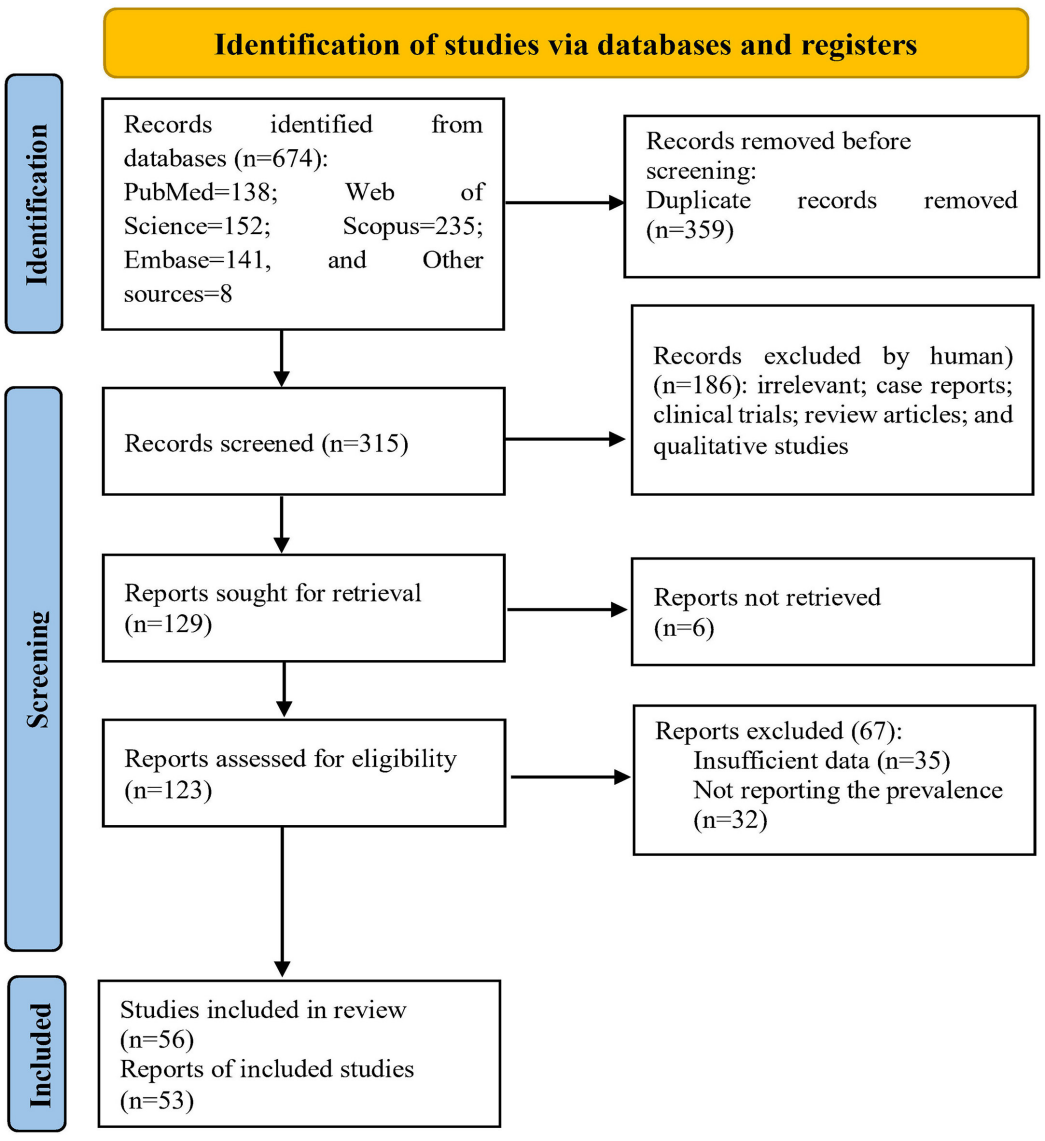
Publication bias.

Also, the prevalence of self-medication in studies collected in 2021 (51.2%, 95% CI: 40.5–61.8) was higher than in studies collected in 2020 (48%, 95% CI: 40.3–55.7). The results of the subgroup analysis are presented in Table 3, the meta-regression results showed that the prevalence of self-medication was not related to the study sample size (*p* = 0.629), and the mean age of participants (*p* = 0.170). Publication bias was also not significant (*p* = 0.363) (Figure 3).

**Table 3:** Prevalence of self-medication during the COVID-19 pandemic by continent, target population, time of data collection.

**Subgroup**		**Number of studies**	**Prevalence (95% CI)**	**Between studies**			**Between subgroups**	
				**I^2^**	**P_heterogeneity_**	**Q**	**Q**	**P_heterogeneity_**
Continent	Asia	31	53 (45–61)	99.40	0.001	11949.77	126.44	0.001
	Europe	3	40.8 (35–46.8)	92.06	0.001	14.43		
	Africa	11	41.5 (29.5–53.5)	99.06	0.001	1160.27		
	America	10	47.8 (33.6–62)	99.79	0.001	6933.03		
Target group	General population	29	49 (41–57)	99.73	0.001	18112.35	5.16	0.397
	Students	11	54.5 (40.8–68.3)	98.89	0.001	946.58		
	Healthcare workers	5	32.5 (16–49)	99.07	0.001	520.00		
	Patients with COVID-19	4	41.7 (25.5–58)	97.72	0.001	195.87		
	Others*	6	53.5 (37.3–69.7)	99.02	0.001	782.70		
Data collection time	2020	31	48 (40.3–55.7)	99.66	0.001	9891.97	23.00	0.891
	2021	16	51.2 (40.5–61.8)	99.34	0.001	2233.33		
	Unknown	8	49.7 (32.4–67)	99.31	0.001	2061.28		

^*^Inflammatory bowel disease patients; dental patients; pregnant and postpartum women, elderly, dermatology patients; faculty employed; and out patients.

## Discussion

This study, which was conducted to estimate the cumulative prevalence of self-medication in the COVID-19 pandemic, showed that nearly half of the people have self-medicated. The results of a previous meta-analysis conducted on 14 relevant studies published between April 1, 2020, and March 31, 2022, showed that the pooled prevalence of self-medication was 44.9% (65). In addition to updating that study, we reported the results of 56 eligible studies by continent, target population, and time of data collection in the present study. James et al. (66) noted that people worldwide practice self-care practices, which in many cases are done through self-medication.

If self-treatment is done correctly, it can be beneficial for one's health. The WHO also recognizes correct self-medication as a type of self-medication (67). Mir et al. (48) mentioned that self-medication encourages patients to rely on themselves in making decisions for the management of minor illnesses.

The highest prevalence of self-medication was related to students. A recent meta-analysis showed that the prevalence of self-medication in students (before the COVID-19 pandemic) was 49.5% (68). The prevalence of self-medication among college students seems to have increased during this pandemic. This finding can be attributed to their higher education level compared to the general population. The lowest prevalence of self-medication was related to healthcare workers. This finding may be due to their familiarity with the consequences of self-medication. The low prevalence of self-medication in the European continent can be due to clear instructions regarding the distribution and provision of medicine and the general knowledge of the people of that region. The prevalence of self-medication was higher in studies that collected data in 2021. The reason for this finding can be the reduction of people's extreme fear of the COVID-19 virus and everyone's familiarity with this disease. False information at the beginning of the disease and the health measures applied by the governments had led to psychological distress and significant fear in the people, so in some countries, people bought and stored toilet paper, face masks and staple foods and even armed (69).

Although the high prevalence of self-medication can indicate people's self-care behavior, it can also lead to serious risks, especially for the elderly, children, pregnant women, and people with underlying diseases. Due to the fact that in the future, it is possible that other waves of the COVID-19 pandemic or other pandemics may occur, it is necessary to prepare and present instructions and guidelines to separate safe self-medication from high-risk ones so that the risks of self-medication are minimized.

## Limitations

One of the limitations of this study was that gray texts were not included in the analysis. The reason was the lack of access to the databases that provided these documents. Another limitation of this study was that in this systematic review and meta-analysis, only articles published in English were analyzed.

## Conclusion

This study showed that self-medication during the COVID-19 pandemic was highly prevalent, so nearly half of the people had self-medicated at this time. The prevalence of self-medication in students was higher than in other groups. Also, with increasing age, the prevalence of self-medication had an almost downward trend. Considering the consequences of self-medication, it seems necessary to educate the general population through the media to increase drug information and improve their health literacy. Awareness of drug risks can reduce the practice of self-medication now and even in pandemics that may occur in the future.

## Data availability statement

The original contributions presented in the study are included in the article/Supplementary material, further inquiries can be directed to the corresponding author.

## Author contributions

GK and FT contributed to designing and performing this systematic review. RG and GK checked the data and conducted data analyses. SA and ML contributed to writing and editing the paper. All authors read and confirmed the final version of the manuscript.

## Conflict of interest

The authors declare that the research was conducted in the absence of any commercial or financial relationships that could be construed as a potential conflict of interest.

## Publisher's note

All claims expressed in this article are solely those of the authors and do not necessarily represent those of their affiliated organizations, or those of the publisher, the editors and the reviewers. Any product that may be evaluated in this article, or claim that may be made by its manufacturer, is not guaranteed or endorsed by the publisher.
