# Supplementary material for: Prevalence of self-medication during COVID-19 pandemic: A systematic review and meta-analysis
Source: Front Public Health. 2022 Nov 3;10:1041695. doi: 10.3389/fpubh.2022.1041695 (PMC9669079; doi:10.3389/fpubh.2022.1041695)
Supplement: Supplementary file 2 [file Table_2.DOCX]

**Supplementary file 2:** The bias assessment of included studies

| Author (year) | Selection (Maximum 5 stars) | | | | Comparability (Maximum 2 stars) | Outcome (Maximum 3 stars) | |
| --- | --- | --- | --- | --- | --- | --- | --- |
|  | Item 1 | Item 2 | Item 3 | Item 4 | Item 5 | Item 6 | Item 7 |
| Alsaad (2022) | 1 | 1 | 0 | 1 | 0 | 2 | 1 |
| Kashyap (2022) | 1 | 1 | 0 | 1 | 0 | 2 | 1 |
| Malik (2022) | 1 | 1 | 0 | 1 | 0 | 2 | 1 |
| Likhar (2022) | 1 | 0 | 0 | 1 | 0 | 1 | 1 |
| Aitafo (2022) | 1 | 1 | 0 | 2 | 0 | 2 | 1 |
| Alavi Namvar (2022) | 1 | 0 | 0 | 1 | 0 | 1 | 1 |
| Barakat (2022) | 1 | 0 | 0 | 1 | 0 | 1 | 1 |
| Yasmin (2022) | 1 | 1 | 1 | 2 | 2 | 1 | 1 |
| Acharya (2022) | 1 | 1 | 1 | 0 | 0 | 1 | 1 |
| Alateeq (2022) | 1 | 1 | 1 | 1 | 2 | 1 | 1 |
| Amuzie (2022) | 1 | 1 | 1 | 2 | 2 | 1 | 1 |
| Bello (2022) | 1 | 0 | 0 | 2 | 0 | 2 | 1 |
| Gaviria-Mendoza (2022) | 0 | 0 | 0 | 2 | 0 | 1 | 1 |
| Dehghan (2022) | 1 | 1 | 1 | 2 | 0 | 2 | 1 |
| Gerbier (2022) | 1 | 1 | 1 | 1 | 1 | 1 | 1 |
| González-González (2022) | 1 | 0 | 0 | 0 | 0 | 2 | 1 |
| Gupta (2022) | 0 | 0 | 0 | 1 | 0 | 2 | 1 |
| Heemskerk (2022) | 1 | 0 | 0 | 1 | 0 | 1 | 1 |
| Joseph (2022) | 1 | 1 | 0 | 0 | 2 | 2 | 1 |
| Kumari (2022) | 1 | 0 | 0 | 1 | 0 | 1 | 1 |
| Mahmoudi (2022) | 1 | 0 | 0 | 2 | 0 | 2 | 1 |
| Odis (2022) | 1 | 1 | 0 | 1 | 0 | 2 | 1 |
| Okoye (2022) | 1 | 1 | 0 | 1 | 2 | 2 | 1 |
| Rojas-Miliano (2022) | 1 | 1 | 0 | 1 | 2 | 2 | 1 |
| Toure (2022) | 0 | 0 | 0 | 1 | 2 | 2 | 1 |
| Vasquez-Elera (2022) | 0 | 0 | 0 | 1 | 2 | 2 | 1 |
| Zeng (2021) | 1 | 0 | 0 | 1 | 0 | 2 | 1 |
| Saleem (2021) | 1 | 1 | 1 | 2 | 0 | 2 | 1 |
| Sadio (2021) | 1 | 1 | 1 | 1 | 0 | 2 | 1 |
| Ruiz-Padilla (2021) | 0 | 0 | 1 | 1 | 2 | 2 | 1 |
| Mota (2021) | 1 | 1 | 1 | 0 | 0 | 2 | 1 |
| Zhang (2021) | 0 | 0 | 0 | 2 | 0 | 2 | 1 |
| Heshmatifar (2021) | 1 | 1 | 0 | 2 | 0 | 2 | 1 |
| Ainsy Goldlin (2021) | 1 | 1 | 0 | 2 | 0 | 1 | 1 |
| Alonso-Castro (2021) | 1 | 1 | 0 | 2 | 0 | 2 | 1 |
| Azhar (2021) | 1 | 0 | 0 | 1 | 0 | 2 | 1 |
| Chopra (2021) | 1 | 0 | 0 | 1 | 0 | 2 | 1 |
| D’arqom (2021) | 1 | 1 | 1 | 1 | 0 | 2 | 1 |
| Faqihi (2021) | 0 | 0 | 0 | 1 | 0 | 2 | 1 |
| Mir (2021) | 1 | 1 | 0 | 1 | 0 | 1 | 1 |
| Islam (2021) | 1 | 1 | 0 | 0 | 2 | 1 | 1 |
| Elayeh (2021) | 1 | 1 | 0 | 2 | 0 | 2 | 1 |
| Choudhary (2021) | 1 | 1 | 0 | 0 | 0 | 2 | 1 |
| Sen Tunc (2021) | 1 | 0 | 1 | 2 | 0 | 2 | 1 |
| Sikdar (2021) | 1 | 1 | 0 | 0 | 0 | 2 | 1 |
| Quispe-Cañari (2021) | 1 | 1 | 1 | 2 | 0 | 2 | 1 |
| Tekeba (2021) | 1 | 1 | 1 | 2 | 2 | 2 | 1 |
| Tobaiqi (2021) | 1 | 0 | 1 | 1 | 0 | 1 | 1 |
| Vinay (2021) | 1 | 0 | 1 | 1 | 0 | 1 | 1 |
| Wegbom (2021) | 1 | 1 | 1 | 2 | 2 | 2 | 1 |
| Onchonga (2020) | 0 | 0 | 1 | 2 | 2 | 2 | 1 |
| Heydargoy (2020) | 0 | 0 | 0 | 1 | 0 | 2 | 1 |
| Makowska (2020) | 1 | 1 | 1 | 0 | 2 | 2 | 1 |
| Mansuri (2020) | 1 | 1 | 0 | 2 | 0 | 1 | 1 |
| Nasir (2020) | 1 | 0 | 0 | 2 | 0 | 1 | 1 |
| Angeles (2020) | 1 | 1 | 1 | 1 | 0 | 1 | 1 |

Item 1-Representativeness of the sample (1 star); Item 2- Sample size (1 star); Item 3- Non-respondents (1 star); Item 4- Risk factors (2 stars); Item 5- Non-respondents (2 stars); Item 6- Assessment of the outcome (2 stars); and Item 7- Statistical test (2 stars).
